# A Biphasic Hydrogel with Self-Healing Properties and a Continuous Layer Structure for Potential Application in Osteochondral Defect Repair

**DOI:** 10.3390/polym15122744

**Published:** 2023-06-20

**Authors:** Yue Jiang, Shanzhu Guo, Jingjing Jiao, Long Li

**Affiliations:** College of Materials and Metallurgy, Guizhou University, Guiyang 550025, China

**Keywords:** biphasic scaffolds, self-healing hydrogel, osteochondral repair

## Abstract

The treatment of osteochondral defects remains challenging due to the limited healing capacity of cartilage and the poor results of traditional methods. Inspired by the structure of natural articular cartilage, we have fabricated a biphasic osteochondral hydrogel scaffold using a Schiff base reaction and a free radical polymerization reaction. Carboxymethyl chitosan (CMCS), oxidized sodium alginate (OSA), and polyacrylamide (PAM) formed a hydrogel (COP) as the cartilage layer, while hydroxyapatite (HAp) was incorporated into the COP hydrogel to obtain a hydrogel (COPH) as an subchondral bone layer. At the same time, hydroxyapatite (HAp) was incorporated into the COP hydrogel to obtain a hydrogel (COPH) as an osteochondral sublayer, combining the two to obtain an integrated scaffold for osteochondral tissue engineering. Interlayer interpenetration through the continuity of the hydrogel substrate and good self-healing properties due to the dynamic imine bonding of the hydrogel resulted in enhanced interlayer bond strength. In addition, in vitro experiments have shown that the hydrogel exhibits good biocompatibility. It shows great potential for osteochondral tissue engineering applications.

## 1. Introduction

Articular cartilage is a specialized connective tissue that covers the ends of the bones in a joint and provides a smooth surface for joint movement, facilitating the transmission of mechanical loads and reducing the coefficient of friction [1]. However, the lack of blood vessels, lymphatic vessels, and nerve tissue and the low number of cells in articular cartilage, result in a limited ability to regenerate cartilage [2]. Damage to cartilage and its degeneration often lead to osteoarthritis, a debilitating disease that affects millions of people worldwide [3,4]. However, current therapeutic approaches to cartilage repair, such as autologous chondrocyte implantation and microfracture, are limited by issues such as limited donor sources and immune reactions, resulting in osteochondral repair remaining a challenge [5,6,7]. Recently, tissue engineering has attracted the attention of a large number of researchers as a promising potential option for repairing osteochondral defects [8]. In addition, a growing number of studies have shown that the treatment of cartilage lesion surfaces is ineffective without the support of an intact subchondral bone [9]. Therefore, total regeneration of the entire osteochondral unit and not just the articular cartilage layer should be considered for effective treatment [10]. Typically, monophasic scaffolds do not mimic the natural environment of osteochondral bone well when used to form new tissue, leading to problems of poor cartilage and subchondral bone fusion and limiting the scope of monophasic scaffolds [11,12]. In contrast, biphasic osteochondral scaffolds are more attractive than monophasic scaffolds because they mimic the cartilage and subchondral bone structure of the entire osteochondral unit, providing a favorable microenvironment which, in turn, promotes the growth of cartilage and subchondral bone areas and shortens the defect repair cycle [13,14]. For example, Wang et al. [15] used an injectable, self-crosslinked mercapturic hyaluronic acid (HA-SH)/collagen type I (Col I) hybrid hydrogel combined with BCP ceramics to fabricate a new bilayer scaffold to mimic the specific structure of osteochondral bone. Although these methods have had some success in osteochondral regeneration, they also have limitations. Some biphasic scaffolds use different primary materials for the cartilage and subchondral bone layers. Some of the biphasic scaffolds used different primary materials, which resulted in a weak connection between the two phases, because there is no continuous phase in the different phases [16]. Since the integration of the cartilaginous and subchondral layers of the biphasic osteochondral scaffold is essential for the construction of an integrated osteochondral scaffold, material properties should be explored to improve the integration of the cartilaginous and subchondral layers [17].

A hydrogel is a soft material with a three-dimensional network structure formed via the cross-linking of covalent and non-covalent bonds which has tunable physical and chemical properties [18]. Hydrogels have a wide range of applications in many fields, particularly in the biomedical field, due to their compositional and structural diversities, which provide them with properties such as environmental responsiveness and high water contents and are very similar to the extracellular matrix (ECM) of natural cartilage, which facilitates cell growth [19]. Recently, self-healing hydrogels based on dynamic covalent bonds (disulfide bonds [20], imine bonds [21], acylhydrazone bonds [22], phenyl boronate ester complexations [23]) and non-covalent bonds (hydrogen bonds [24], host-guest interactions [25], hydrophobic interactions [26], electrostatic interactions [27], and π-π stacking [28]) have received much attention for their ability to maintain the structural integrity of the network. The self-healing hydrogel can achieve external force-free healing, restore mechanical properties, and extend the life of the hydrogel [29]. In these interactions that form self-healing hydrogels, among other things, the hydrogels are self-healing under physiological conditions without the need for additional stimuli, such as an acidic or alkaline environment, which is suitable for tissue engineering [30]. However, the unstable nature of the dynamic bonding leads to the rapid degradation of the hydrogel under physiological conditions, making it difficult to use self-healing hydrogels for osteochondral tissue engineering. Therefore, the three-dimensional network structure of the hydrogel needs to be further enhanced by introducing other stable cross-linked networks [31,32]. In addition, synthetic-polymer-based hydrogels usually have good mechanical properties which are indispensable for the fabrication of tissue engineering scaffolds [33]. However, they mostly do not exhibit good cell affinity and bioactivity [34]. The other category comprises natural-polymer-based hydrogels, including chitosan [35] and sodium alginate hydrogels [36]. These materials show good biocompatibility and degradability, with good osteochondral repair properties. However, a major drawback of these natural-polymer-based hydrogels is their lack of sufficient mechanical strength to meet the mechanical requirements for articular cartilage repair [37]. Therefore, hybrid hydrogels prepared via combining biologically active natural polymers and mechanically tough synthetic polymers are more beneficial for osteochondral repair.

Physiologically, articular cartilage is rich in proteoglycans, collagen, and water, providing a suitable microenvironment for cellular value-adding and providing excellent mechanical properties [38]. Inspired by the complex composition and microstructure of natural articular cartilage, we have developed a double-network hydrogel (COP) consisting of carboxymethyl chitosan (CMCS), oxidized sodium alginate (OSA,) and polyacrylamide (PAM) as a cartilage layer similar to the extracellular matrix (ECM) of natural cartilage by combining dynamic Schiff base reactions and free radical reactions. Meanwhile, hydroxyapatite (HAp) was incorporated into the hydrogel to obtain a subchondral bone layer hydrogel (COPH) for a smooth transition between layers and to enhance continuity in which the HAp enhances not only the mechanical properties of the hydrogel but also the osteoinductive properties of the hydrogel. In addition, the morphology, swelling, degradation, self-healing, and compressive properties of the individual materials were evaluated, as was the overall cytotoxicity of the biphasic scaffold.

## 2. Materials and Methods

### 2.1. Materials

Carboxymethyl chitosan (CMCS), sodium alginate (medium viscosity), ethylene glycol (AR), sodium periodate (AR), hydroxyapatite (HAp), acrylamide (AM), ammonium persulfate (APS), N, N-methylene bisacrylamide (MBA), and tetramethylene ethylenediamine (TMEDA) were purchased from Shanghai Aladdin Biochemical Technology Co., Ltd. (Shanghai, China), and DMSO (AR) was purchased from Tianjin Fuyu Fine Chemical Co., Ltd. (Tianjin, China). The Cell Counting kit-8 (CCK-8) was purchased from Beijing Solarbio Science & Technology Co., Ltd. (Beijing, China). Calcein-AM was obtained from Sigma-Aldrich Co. (St. Louis, MO, USA). MG63 was acquired from the Institute of Biochemistry and Cell Biology (Chinese Academy of Sciences, Shanghai, China). All the cell culture reagents were provided by Gibco Life Technologies Co. (New York, NY, USA) unless otherwise specified.

### 2.2. Synthesis of Oxidized Sodium Alginate (OSA)

The synthesis of OSA was carried out in an aqueous solution at room temperature according to the previously reported method [39,40]. Briefly, sodium alginate (SA) (5.0 g) was dissolved in 450 mL of deionized water at room temperature; then, sodium periodate (2.0 g) was dissolved in 25 mL of deionized water and added to the above sodium alginate solution. The reaction was stopped by adding 3.5 mL of ethylene glycol and stirring for a further 30 min after 24 h of stirring away from light. The reaction mixture was poured into 500 mL of ethanol containing 2.0 g of NaCl. The precipitate was collected via centrifugation to obtain crude OSA. The OSA was purified by dissolving it in deionized water and dialyzing it in a dialysis bag (8000–14,000 D) for 3 days. Finally, pure OSA was obtained via freeze-drying. ^1^HNMR (JEOL JNMECZ-400 MHz, Japan) measurements were used to confirm the successful preparation of OSA.

### 2.3. Preparation of COP and COPH Hydrogels

CMCS and OSA were dissolved in a PBS buffer (pH = 7.4) to obtain a CMCS solution (3%, *w*/*v*) and OSA solution (8%, *w*/*v*) respectively. Then, 2.25 g of AM was added to 5 mL of CMCS solution, and stirring was continued until the solution was clarified. Then, 22.5 mg of APS and 4.5 mg of MBA were added to 10 mL of OSA solution, and after mixing the two parts for 1 min, TMEDA was added, and the mixture was immediately vortexed to obtain a COP hydrogel. For the COPH hydrogels, CMCS and OSA were dissolved in PBS buffer to prepare 3% CMCS and 8% OSA solutions, respectively. Then, HAp (1%, *w*/*v*) was added to the above solutions, respectively, and ultrasonically dispersed well. The other steps of the COPH hydrogel formation process were the same as in the COP hydrogel formation process.

### 2.4. Self-Healing Properties of Hydrogels

The self-healing capacities of the hydrogels were evaluated via macroscopic autonomous healing and a rheological recovery test. The hydrogel samples were cut in half, and the cross sections of the two halves were brought into close contact to observe the self-healing behavior. The cross sections of the two halves were brought into close contact to observe the self-healing behavior. After 2 h, the gel state of the sample was observed.

The healing ability of the hydrogel was evaluated by cutting a dumbbell-shaped hydrogel sample (inner width: 6 mm; length: 25 mm; thickness: 4.5 mm) into two pieces and allowed to reconnect be simply joining the pieces at 37 °C without using any external stimuli. The tensile strength was measured using a universal testing machine after 2 h of hydrogel self-healing. The healing efficiency was defined by the tensile strength of the gel of the self-healing hydrogel versus the original hydrogel [41].

$$\mathrm{Healing}\;\mathrm{efficiency}\;(\%)=\frac{\sigma_{H}}{{\;\sigma }_{O}\;}\times 100\%$$

where σ_H_ and σ_O_ are the tensile stresses of the self-healed hydrogel and original hydrogel, respectively. The average values and errors were calculated from at least three independent samples for each specimen.

### 2.5. Preparation of a Biphasic Osteochondral Hydrogel Scaffold

Firstly, the COPH hydrogel was prepared in a suitably sized mold via the method described above to obtain the subchondral bone layer. Secondly, the COP sol was poured on top of the COPH hydrogel, which had not been fully cross-linked. Finally, a biphasic osteochondral scaffold simulating cartilage and subchondral bone was obtained by waiting a few minutes at room temperature.

### 2.6. Scanning Electron Microscopy

To observe the internal cross-linking states of the hydrogel scaffolds, the morphologies of the COP and COPH hydrogels were studied via scanning electron microscopy. The lyophilized hydrogels were immersed in liquid nitrogen for 2 min, and the hydrogels were then mechanically crushed to form cross-sections. Finally, after spraying gold on the cross section of the hydrogel, the morphologies of the hydrogels were observed via scanning electron microscopy (SEM, Tescan Mira 4, Brno, Czech Republic) by fixing them on the apparatus with conductive adhesive tape.

### 2.7. Swelling Ratios of the Hydrogels

The hydrogels were processed into similar shapes at room temperature, weighed (*m*_0_), and immersed in deionized water (pH = 7.4) until the hydrogels reached solubilization equilibrium. The samples immersed in water were weighed periodically (*m_t_*), taking care to remove excess water from the surface with filter paper. The hydrogel swelling rate was calculated according to the following equation:

$$\mathrm{Swelling}\;\mathrm{rate}\;(\%)=\frac{m_{t}-m_{0}}{m_{0}}\times 100\%$$

### 2.8. Compression Test

The hydrogel was prepared as a cylinder with a diameter of 10 mm and a height of 6 mm, and the compression strength of the hydrogel was tested at room temperature with a universal testing machine. In the compression test, the compression rate was set to 10 mm/min, at least three specimens were used in each group of compression test, and the experimental data were recorded and averaged.

### 2.9. Degradation of the Hydrogel

The in vitro degradation of the stents was examined via enzymatic digestion. In vitro degradation experiments were performed to examine and test the degradation of the COP and COPH hydrogels over a period of 4 weeks. Briefly, first, the initial weights of the freeze-dried hydrogels were recorded as (*w*_0_), and then the freeze-dried hydrogels were immersed in a PBS buffer (3 mL, pH = 7.4) containing 2 mg/mL of lysozyme and placed in a shaker at 37 °C for slow shaking. The degradation solution was changed regularly every week. Next, the hydrogels were removed at specific time points (*n* = 3). Finally, the removed hydrogels were freeze-dried, and their weights (*w_t_*) were recorded. The degradation rate was calculated using the following equation:

$$\mathrm{Degradation}\mathrm{ratio}\;(\%)=\frac{w_{0}-w_{t}}{w_{0}}\times 100\%$$

### 2.10. In Vitro Biocompatibility

The CCK-8 method was used to detect the biocompatibility of the hydrogel leachate on MG63 cells. Briefly, the lyophilized hydrogels were immersed in a 75% ethanol solution and centrifuged to remove air bubbles. They were then repeatedly sterilized with 75% ethanol, rinsed several times with PBS, placed in a biosafety cabinet under UV light, and sterilized by ozonation. Next, 0.2 g of sterile hydrogel was incubated in 12 mL of MEM (containing NEAA), a medium containing 10% (*v*/*v*) fetal bovine serum, for 7 days at 37 °C. The leachate was filtered through a 0.22 μm nylon membrane to remove impurities. An MG63 cell suspension (10^4^ cells/mL, 200 μL) was inserted into 24-well microplates and incubated at 37 °C for 4 h, and then 300 μL of hydrogel leachate was added and incubated for 5 days at 37 °C in a 5% CO_2_ incubator. The cell viability was measured using a CCK-8 reagent (CCK-8, Beijing Solarbio Science & Technology Co. Ltd., China; CCK-8-to-culture-medium volume ratio of 1/10). The absorbance values were recorded at 450 nm on days 1, 3, 5, and 7 using an enzyme marker (Epoch, Biotek, Winooski, VT, USA).

### 2.11. Live Staining

After inoculating the cells for 1, 3, 5, and 7 days as described above, all samples were washed with PBS solution and stained with Calcein-AM (Beijing Solarbio Science & Technology Co., Ltd., Beijing, China) was added and incubated with the samples in the dark of the incubator for 15 min. After rinsing them with PBS three times, the stained samples were observed via inverted fluorescence microscopy (IX73 + DP80, Olympus, Japan).

### 2.12. Statistical Analysis

All the results were obtained at least in triplicate. Comparisons were performed using a one-way analysis of variance (ANOVA). The minimum significance level was set as * *p* < 0.05, ** *p* < 0.01 and *** *p* < 0.001.

## 3. Results and Discussion

### 3.1. Preparation of COP and COPH Hydrogels

**Figure 1:**
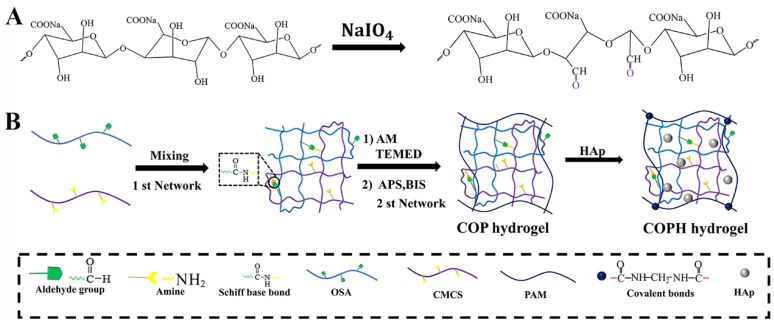
Synthesis and fabrication of the hydrogel. (**A**) Schematic diagram of the synthesis of OSA; (**B**) Schematic diagram of the preparation of the COP and COPH hydrogels.

**Figure 2:**
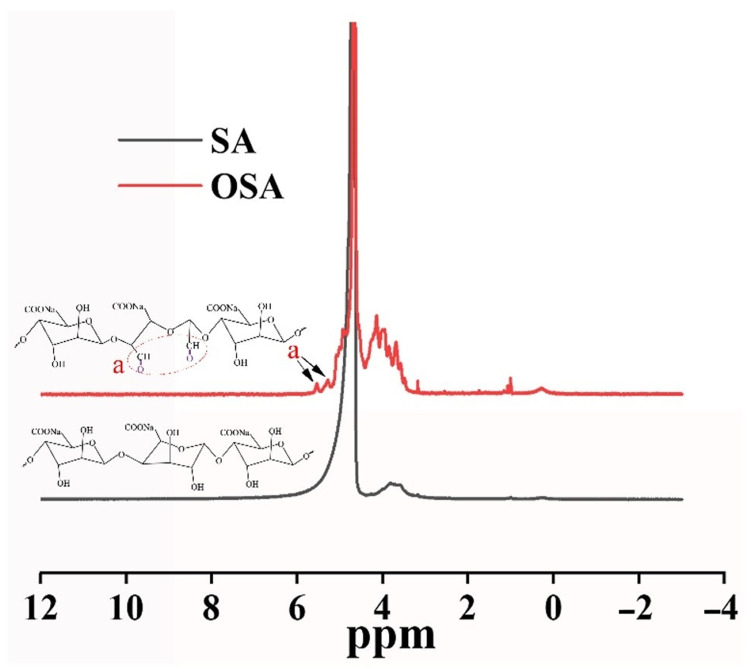
^1^HNMR spectra of OSA and SA.

Chitosan (CS) is considered more advantageous for cartilage tissue engineering applications because of its hydrogel-forming properties, which are due to its rich amine content and its structural similarity to glycosaminoglycans (GAGs) [42]. Furthermore, we oxidized the diol structure adjacent to the SA sugar chain with NaIO_4_, which broke the carbon–carbon single bond and attached it to the adjacent hydroxyl group. Adjacent hydroxyl groups were oxidized to form aldehyde groups, which modified the SA polymer to the OSA. The specific synthesis route is shown in Figure 1A. In this study, ^1^HNMR was used to analyze the chemically modified structure of the OSA. By comparing the ^1^HNMR spectra of the SA and OSA (Figure 2), we could identify the characteristic absorption peak of single-bond -CHO at δ = 5.0–5.6 ppm, and new characteristic peaks at 5.31 ppm and 5.60 ppm, both of which correspond to methine protons, were observed in the OSA, confirming the oxidation of SA [43,44]. Based on the above experimental results, we can be confirmed that the polymers of OSA have been successfully prepared. Due to the Schiff base reaction between the amine group on the CMCS, the aldehyde group on the OSA, and the radical polymerization reaction between the AMs, both the COP and COPH hydrogels could be obtained within 2 min (Figure 1B). The double-network hydrogel was formed in one step in which the first network and the second network in the hydrogel are formed simultaneously without any influence on each other yet stabilize the hydrogel structure together. The incorporation of HAp not only enhances the hydrogel’s 3D network but also induces mineralization to the osteochondral scaffold due to HAp’s high contents of calcium and phosphorus, thus promoting osteoinductive and osteoconductive properties [45].

### 3.2. Microstructure Characterization of Hydrogels

**Figure 3:**
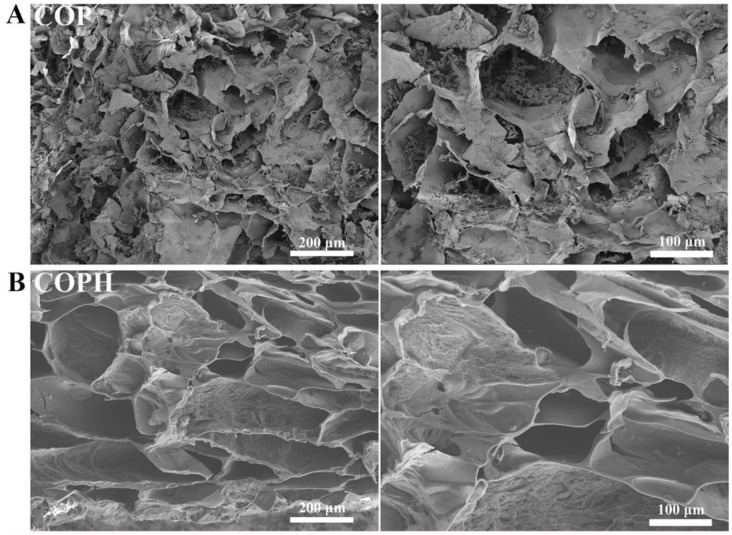
Internal microstructures of hydrogels: (**A**) COP hydrogel; (**B**) COPH hydrogel.

As highly hydrated 3D networks, hydrogels have similar structures and functions to the ECM [46]. The cross-sectional microstructures of the COP hydrogel and COPH hydrogel were examined via SEM. As shown in Figure 3A,B, the partial pore collapse of the COP hydrogel and the good pore structure of the COPH hydrogel could be observed via scanning electron microscopy. In general, both hydrogel scaffolds showed a microporous network structure similar to those of conventional hydrogels; the structure of COPH hydrogels did not change significantly by the addition of HAp, probably due to the lower HAp content. In addition, hydrogels have interconnected, porous microstructures that facilitate the exchange of nutrients and oxygen, which can accelerate the healing of defective tissues [47,48].

### 3.3. Self-healing Performance

**Figure 4:**
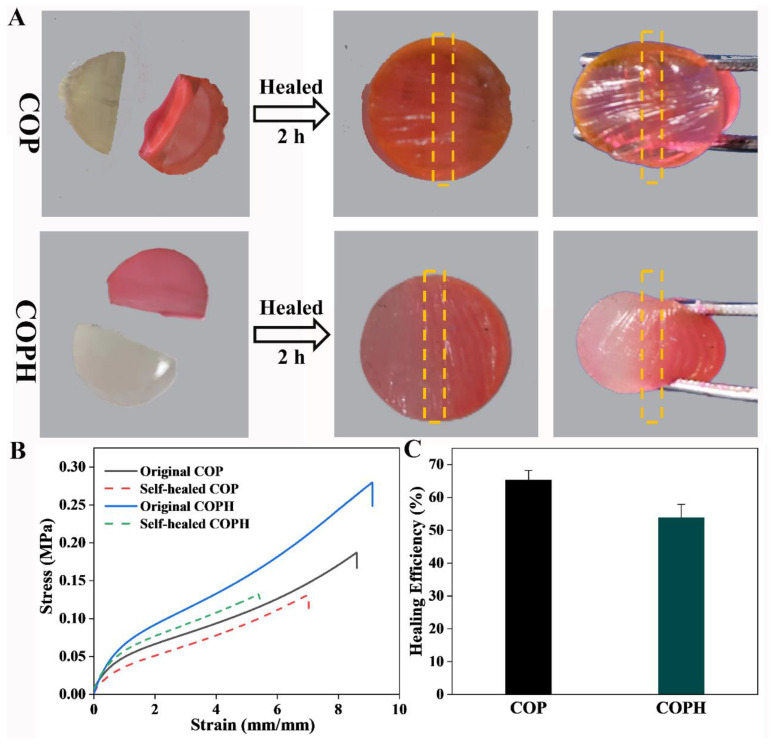
The self-healing properties of COP and COPH hydrogels. (**A**) Macroscopic self-healing properties; (**B**) tensile stress–strain curves of the original and corresponding healed hydrogels; (**C**) healing efficiencies of hydrogels.

In experiments testing the self-healing properties of the hydrogels, OSA was used to react with the Schiff base of CMCS to generate dynamic, reversible imine bonds that conferred self-healing properties to the hydrogels, and the macroscopic self-healing properties of the hydrogels were assessed via direct observation. As shown in Figure 4, the COP and COPH hydrogels were divided into two halves. One half was stained with rhodamine 6G, and the two halves were then gently laminated together. After 2 h, it was observed that the hydrogels were obviously bonded together without cracks, the unstained parts also turned red due to diffusion, and no rupture occurred when squeezed, indicating that the hydrogels had good self-healing properties and repaired the 3D network structure to a large extent, maintaining its structural integrity. In general, in the COP and COPH hydrogels, on one hand, the decoupling and recoupling of the imine bonds obtained from the amino group of the CMCS and the aldehyde group of the OSA via the Schiff base reaction are in dynamic equilibrium. This equilibrium is broken when new surfaces appear on the hydrogel, generating large numbers of aldehyde and amino groups. When the new surfaces come into contact with each other, the imine bonds will be rebuilt, and this process will lead to self-healing behavior. On the other hand, the presence of a large number of hydrogen bonds between PAMs also enhances the self-healing properties to a certain extent. 

Figure 4B shows that the self-healing hydrogel shows a similar curve to the original hydrogel; however it fractures earlier. In addition, probably due to the toughening of HAp as an inorganic filler, the COPH hydrogel has a greater tensile strength. After hydrogel repair, both the tensile stress and tensile strain were reduced, and the COPH hydrogel reduced more compared to the COP hydrogel. If we define the healing efficiency of a hydrogel as the tensile stress of a self-healing hydrogel at fracture divided by the tensile stress of the original hydrogel at fracture, the healing efficiencies of the COP hydrogel and COPH hydrogel are 65.29% and 53.86%, respectively (Figure 4C); the healing efficiency of the COPH hydrogel is lower than that of the COP hydrogel. It may be due to the fact that although HAp enhances the strength of the hydrogel, the HAp in the COPH hydrogels hinders the movement of molecular chain segments, thus reducing the opportunity for amino and aldehyde groups to come into contact with each other in the hydrogel and thus leading to a decrease in the self-healing efficiency of the COPH hydrogels.

### 3.4. Compression Test

**Figure 5:**
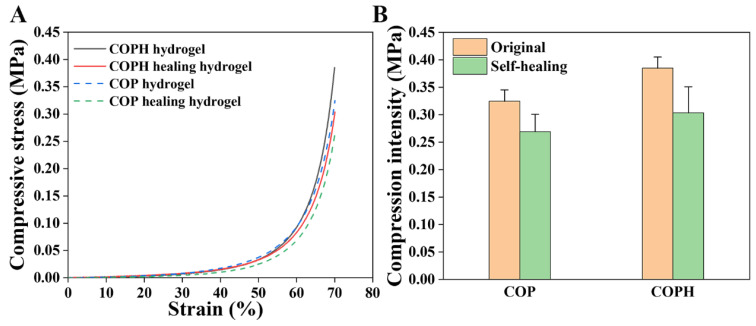
Schematic diagram of mechanical properties of the hydrogel. (**A**) Stress–strain curves; (**B**) compressive intensity.

Stress–strain compression tests were performed to determine the efficiency of self-repair. To protect the instrument, all samples did not fracture when the compressive strain reached 70%, so we defined the compressive stress at 70% of the compressive strain as the maximum compressive stress. It can be seen from Figure 5 that the stress–strain curves of the hydrogels after self-healing are slightly lower than those of the original hydrogels for both the COP hydrogels and COPH hydrogels, indicating that it is difficult to fully recover the mechanical properties of the hydrogels after self-healing. A possible reason for this is that even if the first network consisting of dynamic imine bonds is restored, the second network consisting of polyacrylamide is difficult fully restore.

### 3.5. Swelling of the Hydrogel

**Figure 6:**
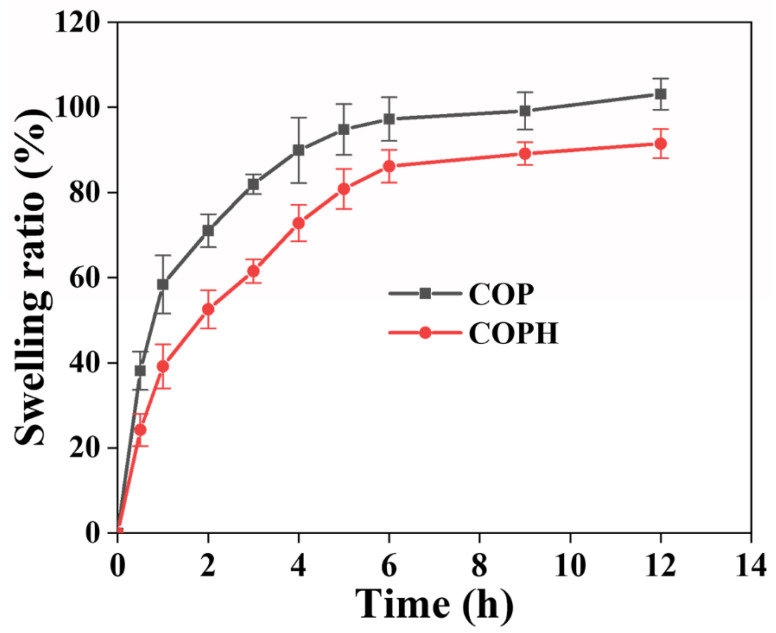
Swelling behaviors of hydrogels.

The swelling property of a hydrogel is an important index of the hydrogel’s performance that can reveal the internal structure of the hydrogel. The swelling ratios of the COPH hydrogel and COP hydrogel were much smaller than that of the PAM hydrogel, as tested by soaking the hydrogels in deionized water. As can be seen from Figure 6, the increases in the swelling rates of both hydrogels in the early stages of swelling are large, indicating more water absorption, which is due to the fact that both hydrogels are composed of hydrophilic polymers. The decrease in hydrogel swelling after the addition of HAp may be because with the addition of HAp, which then occupies some of the pores of the three-dimensional network of the hydrogel, it becomes more difficult for water molecules to enter the interior of the hydrogel, thus leading to a decrease in the swelling of the hydrogel. In addition, hydrogels can hold large amounts of tissue fluid and nutrients in the body and can provide a suitable environment for the cells and tissues that grow into the hydrogel.

### 3.6. Hydrogel Degradation Properties

**Figure 7:**
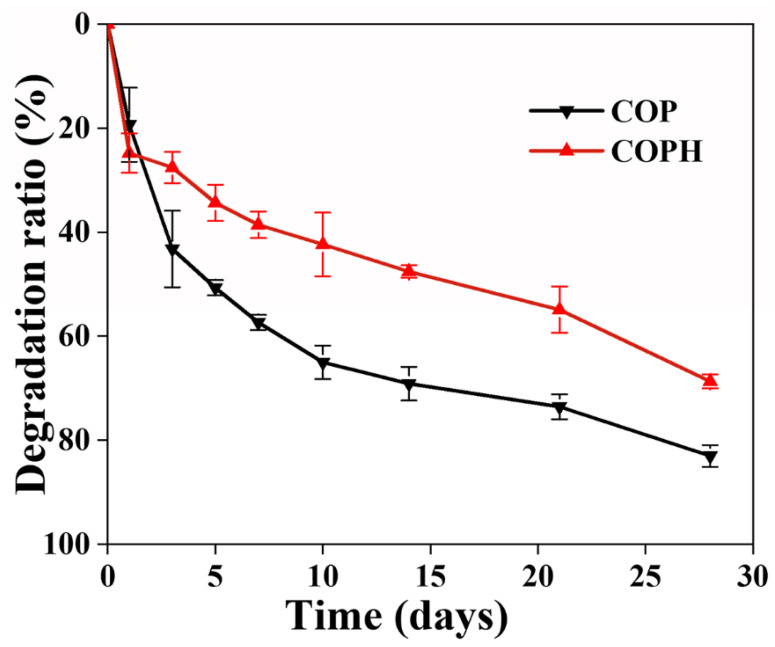
Hydrogel degradation curves.

The degradability of the scaffold is one of the factors that are crucial in evaluating the performance of the scaffold. The ideal tissue-engineered scaffold should degrade at a rate consistent with the rate of new tissue formation, with gradual regeneration of new tissue while the scaffold degrades, continuously providing space for cell and tissue regeneration. From Figure 7, it can be seen that both hydrogels degraded, and their degradation rates were faster in the first three days. The possible reason is that the cross-linking rate is too fast resulting in some of the polymers in the hydrogel not being cross-linked, thus leading to a faster degradation rate of COP hydrogel, which reached 82.96% ± 2.08% at day 28.It can be seen that the degradation of the hydrogels decreased with the addition of HAp to the hydrogel degradation rate. This may be due to the lower crosslinking density of the hydrogels without the addition of HAp and the insufficient linkage between the internal networks, which make them more susceptible to degradation under the action of water solubilization. This also indicates that a higher crosslinking density is beneficial in improving the resistance to hydrolysis. This conclusion is verified by the hydrogel’s mechanical properties. In addition, the natural polymers CMCS and OSA are more easily degraded by enzymes, and the degradation rates of PAM and HAp, are slower, so the overall degradation rate of the COPH hydrogels was lower than that of the COP hydrogels.

### 3.7. Cell Compatibility Evaluation of Hydrogels

**Figure 8:**
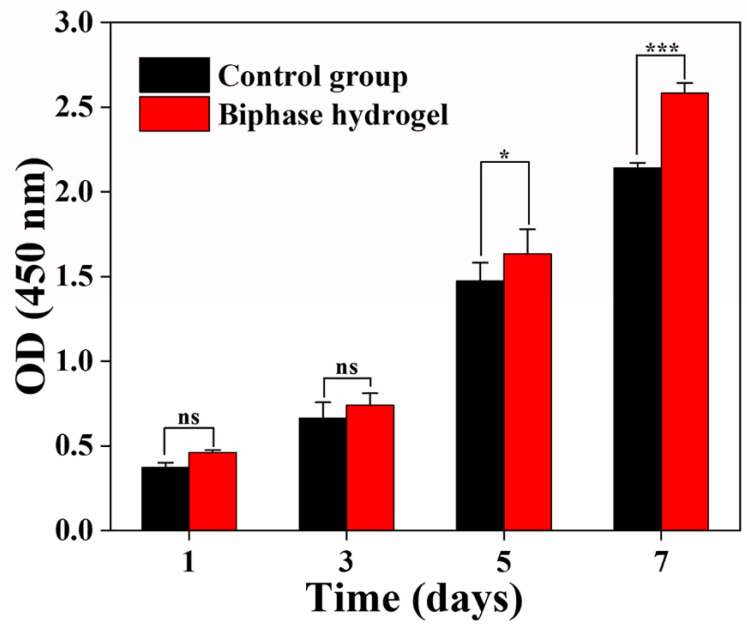
Cell viability at days 1, 3, 5, and 7, detected via CCK-8 assay.

**Figure 9:**
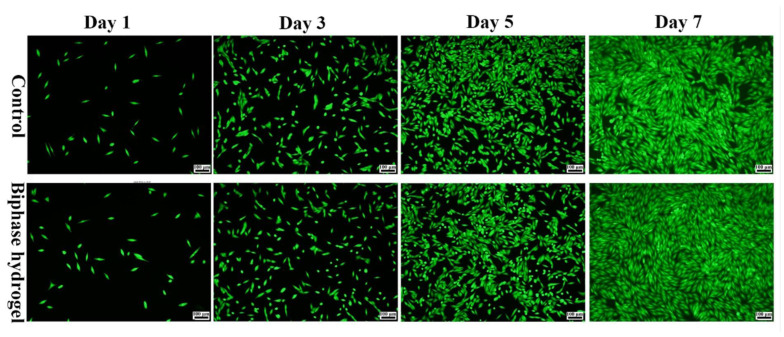
Live cell staining of hydrogel leachate.

Tissue-engineered scaffolds should have good cytocompatibility to better support cell growth and proliferation in vitro. It is well known that both CMCS and OSA, which are biodegradable and biocompatible, have the ability to promote tissue healing. It has been reported that CMCS/OHA degradable, chitosan-based hydrogels were prepared with CMCS as the backbone and oxidized hyaluronic acid as the cross-linking agent, which has appreciable hemostatic properties with good biodegradability and biocompatibility [49]. The cell proliferation results showed no significant difference between the hydrogel and control groups on the first and third days. On day 5, the proliferation of MG63 in the hydrogel leachate was significantly better than that of the control. In particular, on day 7, the proliferation rate of MG63 in the hydrogel leachate was significantly higher than that of the control. It was shown that the hydrogel component can promote cell proliferation, the hydrogel has good biocompatibility to promote the proliferation of MG63, and the bionic biphasic hydrogel has good biocompatibility to MG63 (Figure 8). To further visualize the proliferation of MG63 cells in the hydrogel leachate, we observed by staining live cells with Calcein-AM. The status of the MG63 cells cultured in the hydrogel leachate was observed via fluorescence microscopy (Figure 9), and the cell fluorescence images showed a shuttle-shaped morphology similar to that of the control. Almost all cells were stained green with live cells, indicating that the MG63 cells in the hydrogel leachate were numerous and proliferating at the same time. This shows that the hydrogel has good biocompatibility, which is consistent with the cell proliferation results. It was also further demonstrated that the biphasic hydrogel scaffold had good cytocompatibility. In conclusion, the biphasic bionic hydrogel scaffold prepared in this study has good cytocompatibility and can be used for cartilage repair and tissue regeneration.

## 4. Conclusions

In this study, we developed a biphasic osteochondral hydrogel scaffold to enhance the interface between the two layers within the osteochondral scaffold by increasing the continuity of the cartilage layer with the subchondral bone layer substrate and the self-healing properties of the hydrogel to address the current problem of the easy delamination of the biphasic scaffold. The hydrogel has good self-healing properties which not only prolong the life of the hydrogel but also allow the biphasic hydrogel layers to recover even if they are damaged. In addition, the biphasic osteochondral scaffold was shown to be biocompatible and non-toxic to cells by cell proliferation results and fluorescence staining. Therefore, this biphasic hydrogel may be a promising candidate material for osteochondral tissue engineering.

## Data Availability

Not applicable.
